# Redefining cancer research for therapeutic breakthroughs

**DOI:** 10.1038/s41416-024-02634-6

**Published:** 2024-02-29

**Authors:** Arseniy E. Yuzhalin

**Affiliations:** Department of Molecular and Cellular Oncology, The University of Texas MD Anderson Cancer Center, Houston, TX 77030 USA

**Keywords:** Policy, Funding, Cancer, Drug development

## Abstract

Cancer research has played a pivotal role in improving patient outcomes. However, despite the significant investment in fundamental cancer research over the past few decades, the translation of funding into substantial advancements in cancer treatment has been limited. This perspective article employs a detailed analysis to outline strategies for promoting innovation and facilitating discoveries within the field of cancer research.

Cancer research is among the most heavily funded areas of science. Thousands of original research articles on cancer are published annually; many of these propose novel mechanisms of tumor growth, migration, metastasis, survival, immune escape etc. Many critics have questioned the validity and necessity of such studies, raising concerns over mass generation of irreproducible, redundant, and unjustified research, both basic and clinical [1–5]. Remarkably, the three primary pillars of cancer treatment—surgery, chemotherapy, and radiotherapy—which were actively employed at least 60 years ago, continue to serve as the key treatment options today. We have witnessed a dramatic rise in the survival rates of patients undergoing treatment with chemotherapy and radiotherapy. However, these improvements have primarily stemmed from the development of more effective chemotherapy agents [6] and their combinations [7, 8] and the evolution of radiotherapy approaches, owing to significant advancements in technology. Earlier diagnosis, improved stratification and better management of treatments have further contributed to improved survival. Certainly, novel targeted therapies and immunotherapies have provided great benefits for cancer patients in recent decades. Unfortunately, these treatments are burdened with multiple pitfalls, including: (i) their applicability being restricted to a specific molecular subset of cancer patients, (ii) exorbitant costs, (iii) limited availability, and (iv) tumors’ developing resistance to therapy. Indeed, only ~40% of cancer patients in the United States were eligible to receive immune checkpoint inhibitors in 2018, and only 12.46% of these patients responded to therapy [9]. The cost of these therapies presents another critical issue. For example, the total treatment expense for chimeric antigen receptor T-cell therapy has been estimated to reach up to $500,000 for patients experiencing severe cytokine release syndrome, which is a common adverse reaction to this type of treatment [10]. Furthermore, the availability of targeted therapies and immunotherapies in low- and middle-income countries is minimal [11]; thus, 85% of the world’s population still relies on surgery, chemotherapy, and radiotherapy for cancer treatment. Additionally, while most targeted therapies and immunotherapies extend a patient’s survival, they are rarely, if ever, 100% curative without surgery and/or intensive chemo-/radiotherapy regimens. Resistance to targeted therapy is frequently inevitable and leads to tumor recurrence in most patients [12]. For example, BRAF-targeting drugs radically changed the clinical practice in melanoma treatment, extending median survival from 6 to 30 months [13]. However, most patients with BRAF-mutated metastatic melanoma experience a relapse in the first year after targeted therapy discontinuation, of which 50% occur in the first 3 months [14]. Clearly, innovative approaches for cancer treatment are urgently needed. Thus, it would be valuable to explore the reasons behind the stumbling progress of innovative cancer research and speculate on how we should approach cancer research in the next decades, given the constraints of climate change, limited economic growth and geopolitical turbulence.

Evidence-based medicine has flourished due to the application of methodological reductionism, an approach that would break down complex phenomena into smaller units which could be comprehended separately, thus making an entire system easier to understand (Fig. 1a). Indeed, reducing the number of variables in complex systems such as the human body would enable the identification of critical aspects regarding the etiology and pathogenesis of diseases. A cornerstone of methodological reductionism and evidence-based medicine is randomized clinical trials, where interventions are broken down into specific components to evaluate their individual effects [15]. Methodological reductionism is also applied in meta-analyses and pharmacological, pre-clinical and diagnostic studies. While methodological reductionism is immensely useful for understanding diseases defined by a single parameter, such as ß-thalassemia, sickle cell disease, hereditary spherocytosis, Fanconi anemia, and other genetic disorders caused by one mutation in one gene [16], it does not enable full explanations of multifactorial disorders such as schizophrenia and cancer [17]. Such illnesses are characterized by multiple layers of complexity, and the ability to capture this complexity is limited by (a) possibilities of current technology and (b) inability to recognize possible interactions between individual units of a complex system (Fig. 1b, c). In addition, in-depth understanding of systems’ fragments may not always accurately predict the behavior of the entire system. Thus, whereas methodological reductionism was extremely useful in the early stage of scientific knowledge, it proves itself less effective when we reach a general ontological consensus on the nature of things, including diseases. The remaining gaps in the picture are immensely difficult to fill in by simply dissecting them into even smaller units and investigating them separately.

**Fig. 1:**
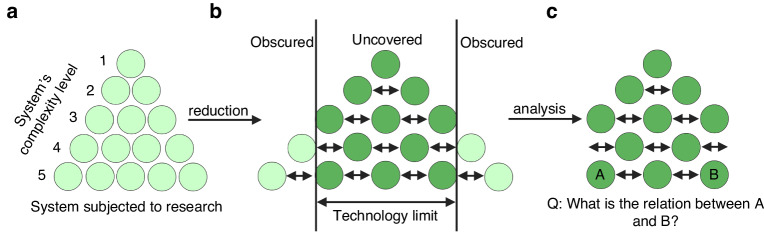
Reductionism overview. **a** Methodological reductionism views complex entities as a sum of smaller components, with the complexity of any system being dependent on the number of smaller units comprising the whole. The complexity of any system is dependent on the number of smaller units composing the whole. **b** Breaking down a complex system into smaller parts facilitates better understanding. However, technological limitations may prevent us from fully comprehending the entire system. **c** While individual parts of a complex system can be thoroughly investigated, the interactions between certain components, as well as the functioning of the system as a whole, often remain unclear.

Human health is inherently holistic in nature, and cancer is one of the most complex diseases. An alternative approach—holism—has recently became popular in cancer research, soon after advent of systems biology at the beginning of 21st century [18]. In contrast to reductionism, holism emphasizes studying complex systems as integrated wholes rather than breaking them down into individual components [19]. A benefit of this approach is evident: it enables researchers to grasp the intricate connections and relationships within a system, providing a more complete and nuanced understanding of the subject matter. The holistic approach in cancer research is evident in single-cell sequencing studies, which offer insights into the clonal evolution and intratumoral heterogeneity of both primary and metastatic tumors [20]. Additionally, although to a lesser extent, these studies shed light on premalignant diseases and the tumor microenvironment [20]. Typically, such studies generate a large amount of big data, including cell type identification, cell state analysis, gene signature and pathway analysis, expression subtype analysis, tumor lineage inference, etc [21]. As a result, we now have access to spatially resolved single-cell atlases of multiple tumor types—a great achievement that, nevertheless, has not yet translated into effective therapies. It is important to note that holistic approaches also have multiple drawbacks, including difficulty in identifying key factors responsible for particular outcomes, inability to perform well-controlled experiments, and limited generalizability because each holistic study is still unique to its specific setting.

Despite the skyrocketing number of targeted therapy drug approvals in the 21st century [22] (Fig. 2a), the 5-year cancer survival rate remained stagnant during 2000–2014 compared with the period of 1975–1999 (Fig. 2b). More recent statistics [23] indicate that the total number of cancer deaths has been rising each year since 1975 up until now (Fig. 2c), even though many deaths have been averted. The level of effort and resources allocated to cancer research may not necessarily translate directly into improved cancer survival rates, particularly in the case of difficult-to-treat cancers like glioblastoma or pancreatic ductal adenocarcinoma. One major factor contributing to rapid growth of cancer incidence and mortality worldwide is aging and growth of the population. Indeed, a recent analysis predicts a continued rise in cancer incidence in the next decades across all economies [24], despite significant efforts to promote early diagnosis tools [25] and develop cancer vaccines [26].

**Fig. 2:**
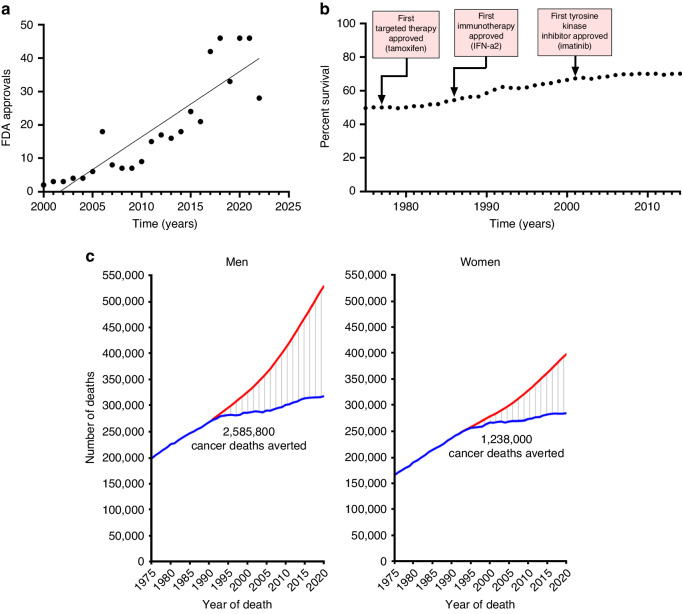
Ending cancer: are we there yet? **a** Rate of targeted therapy and immunotherapy oncology drug approvals by the FDA during 2000-2022 (cytotoxic drugs excluded). Source: analysis of dataset from Scott et al. 2023. **b** 5-year relative survival from all cancer types in the U.S. during 1975–2014. Source: SEER Program, National Cancer Institute. **c** Total number of cancer deaths (blue) in the U.S. and number of cancer deaths that would have been expected if cancer death rates had remained at their peak (red) (Reproduced from Figure 8 in Siegel et al. [23], under the (Creative Commons Attribution-NonCommercial-NoDerivs, https://creativecommons.org/licenses/by-nc-nd/4.0/).

Another reason behind this could be a steady decline in the disruptiveness of science and technology over the past few decades [27]. Across all domains of science, research is generating less progress despite the strong growth in production of papers. This increase in publication is accompanied by reduced novelty of studies, increased self-citations, and more importantly, dominant influence of “superreductionism”, in which papers and patents are using narrower and narrower portions of existing knowledge [27]. Indeed, recent findings in cancer research often explain multifaceted tumor phenotypes, such as therapy resistance, metastatic potential, dormancy, etc., through subtle alterations of a single gene or protein, or even posttranslational modifications of a specific amino acid in a protein sequence. Achieving such significant causal relationships may be possible while using overly simplified systems in the laboratory; however, they are unlikely to hold significant meaning in real-life scenarios of human disease. It is likely that we are either reaching or have already reached the carrying capacity for disruptive science, where there is an extremely high knowledge burden and a deeper understanding of the peculiarities and nuances of known phenomena, but the dearth of groundbreaking “game changer” discoveries (Fig. 3). If true, this would enforce a paradigm shift for stakeholders and academia leaders determined to radically increase survival and quality of life of patients with cancer.

**Fig. 3:**
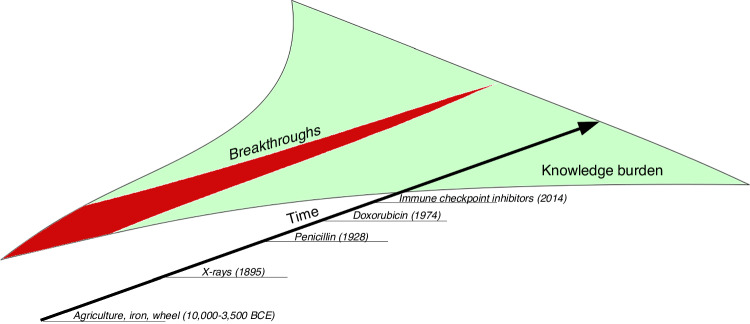
Relationship between disruptive science and knowledge burden. Breakthroughs are becoming less frequent while our knowledge of the world is rapidly expanding. The timeline is arbitrary.

The question arises: how should we approach cancer research in the coming decades to enhance innovation and foster disruptive discoveries, which will ultimately bring therapeutic breakthroughs? Several avenues for improvement should be explored:

- We should refrain from adopting a super reductionist approach in research and instead look for a wise balance between reductionism and holism when designing research projects. Furthermore, mechanistic studies should not be prioritized over functional studies. Whereas plenty of drugs have an incompletely defined mechanism of action (e.g., paracetamol, metformin, lithium), their importance is difficult to overestimate. A recent study found that off-target toxicity is a common mechanism of action of cancer drugs undergoing clinical trials [28]. This finding potentially explains why many cancer drugs fail in clinical trials, emphasizing the complexity of real-life scenarios that often surpass our initial expectations. While investigating the mechanism of action remains extremely important, a holistic understanding of the intricate interactions within biological systems is essential for comprehensively addressing the challenges in drug development;
- While incremental knowledge is useful, only high-risk/high-reward approaches can lead to disruptive science. Nonetheless, the risks associated with high-risk/high-reward projects should be carefully managed;
- Supercollaborations among multiple international groups, each possessing unique expertise and adopting an implementation-oriented approach, are necessary to maximize knowledge synthesis and achieve breakthroughs. The recently introduced CRUK-NCI Cancer Grand Challenge [29] initiative stands as a good example of such an approach. However, it is unfortunate that some of the declared challenges in this program hold more epistemological value rather than practical significance (e.g., e-cigarettes and cancer, aging and cancer, etc.). Another notable example is the Cancer Moonshot initiative [30], which started in 2016 to reduce the cancer death rate by half within 25 years. In its first four years, the initiative led to the start of 49 clinical trials and more than 30 patent fillings [31]. However, the actual impact on survival and quality of life remains to be thoroughly assessed. It is crucial to evaluate the success of super collaborations by examining concrete improvements in real-world patient outcomes;
- When designing research projects, proteomics approaches should always take precedence over transcriptomics, as proteins serve as the ultimate functional units, whereas mRNA is transient and susceptible to degradation. Unfortunately, transcriptomics is more commonly used in cancer research, whereas proteomics is often neglected in functional screens and other high-throughput studies. In addition, most targeted therapy drugs target proteins rather than genes; therefore, prioritizing proteomics enables the identification of potential therapeutic targets and biomarkers at the protein level, which is crucial for drug development and personalized medicine;
- We should foster data transparency. For research manuscripts, the practice of sharing raw data should not be limited solely to high-throughput analyses like transcriptomics and proteomics. It should encompass all the raw data used in creating the research manuscript. Unfortunately, very few journals currently enforce such a requirement, leaving a significant room for improvement in this aspect;
- The peer review process should undergo transformation. Traditional peer review is an inefficient instrument for assessing research quality, often criticized for bias, subjectivity, inconsistency, and the pressure to publish positive results, while also lacking the ability to detect misconduct [32, 33]. Reviewers face limitations in time, resources, niche expertise, and may encounter conflicts of interest, hindering impartial and thorough manuscript evaluation. Additionally, authors from less well-reputable institutions or non-Western countries have much fewer opportunities for networking and collaboration with established researchers, which impacts their visibility within the academic community and greatly reduces the likelihood of their work being accepted by reputable journals. Such conservatism often restricts innovative or unconventional ideas, potentially stifling the progress of scientific discovery. Notably, about two-thirds of Nature referees support exploring alternative publication models [34]. Therefore, it would be beneficial for all leading cancer research journals to adopt the following practices: (i) strongly encouraging the deposition and peer review of preprints (e.g., BioRxiv); (ii) rejecting the option to suggest reviewers during manuscript submission to prevent reviewer nepotism; (iii) conducting initial peer review in a double-blind manner (unless the submitted manuscript has a link to a preprint); and (iv) post-acceptance, disclosing the identities of reviewers and their review reports as part of a commitment to transparency;
- The established *modus operandi* of research funding, where publications lead to grants, which, in turn, lead to more publications, does not incentivize result-oriented research. Instead, it often encourages redundant studies that do not lead to “quantum leap” discoveries. An alternative approach can be observed from the COVID-19 experience, where an unprecedented mobilization of resources led to the rapid development of effective vaccines in just around 6 months, as opposed to the typical timeline of 10–15 years. This accomplishment was achieved by a relatively small number of highly efficient research groups, some of which implemented conceptually new (i.e., disruptive) strategies, such as mRNA vaccine technology. It is possible that centralized and focused efforts—possibly controlled at the level of state—with clearly defined goals and direct responsibility of key personnel, may lead to qualitative changes in cancer treatment. When shortlisting research groups for this endeavor, priority should be given to those with real, proven results (e.g., effective therapy developed in the past), rather than focusing on scientometrics or publications in top journals. A selected handful of these groups should receive unconditional resources and undergo external annual oversight. These few groups must be perpetually contracted, freed from grant writing and publishing, focused on developing treatment new regimens or vaccines for the most lethal cancers, prioritizing translational relevance over molecular mechanisms. Initial hypothesis-making will be collegiately conducted by several senior leaders of the project team. The expected result will be the rapid clinical translation of these findings, smoothly conducted by a liaised hospital with a large patient base, and the ultimate efficacy of the team will be evaluated by the improved patients’ survival and quality of life. Unlike big pharma or biotech companies, these groups will be inspired by eradicating the disease rather than financial profit.

Hopefully, future generations will achieve our society’s common goal of completely eradicating cancer-associated mortality and disability.
